# Preoperative Waiting Time Affects the Length of Stay of Patients Treated via Laparoscopic Cholecystectomy in an Acute Care Surgical Setting

**DOI:** 10.3390/jcm13237263

**Published:** 2024-11-29

**Authors:** Livia Bressan, Matteo Maria Cimino, Federica Vaccari, Eugenia Capozzela, Alan Biloslavo, Matteo Porta, Marina Bortul, Hayato Kurihara

**Affiliations:** 1 Department of Emergency Surgery, Fondazione IRCCS Ca’ Granda Ospedale Maggiore Policlinico, Via Francesco Sforza 35, 20122 Milano, Italy; 2 Department of General Surgery, Cattinara University Hospital, Azienda Sanitaria Universitaria Giuliano Isontina (ASU GI), Trieste University, Strada di Fiume 447, 34149 Trieste, Italy

**Keywords:** acute cholecystitis, laparoscopic cholecystectomy, acute biliary pancreatitis, choledocholithiasis, early cholecystectomy, postoperative complications, postoperative length of stay, Clavien–Dindo classification

## Abstract

**Background/Objectives:** Acute cholecystitis (AC) presents a significant burden in emergency surgical settings. Early laparoscopic cholecystectomy (ELC) is the standard of care for AC, yet its implementation varies. This study aims to assess the impact of preoperative waiting time (WT) on postoperative length of stay (LOS) in patients undergoing urgent cholecystectomy. **Methods:** From June 2021 to September 2022, data on patients undergoing urgent cholecystectomy for AC or pancreatitis were collected from two university hospitals. Patients were categorized into early (ELC) or delayed (DLC) cholecystectomy groups based on WT. The primary outcome was the assessment of the variables influencing LOS via univariate and multivariate analyses. **Results:** This study included 170 patients, predominantly female, with a median age of 64.50 years. ELC was performed in 58.2% of cases, with a median WT of 0 days, while DLC was performed in 41.8%, with a median WT of 3 days. Postoperative complications occurred in 21.8% of cases, with LOS being significantly shorter in the ELC group (median 5 days vs. 9 days; *p* = 0.001). Multivariate analysis confirmed that WT (OR 8.08 (1.65–77.18; *p* = 0.033)) was the most important predictor of LOS. **Conclusions:** ELC is associated with a shorter LOS and with DLC, aligning with the WSES recommendations. Earlier surgery reduces the risk of complications and overall hospital costs. An extended WT contributes to a prolonged LOS, underscoring the importance of timely access to operating theaters for acute biliary pathologies.

## 1. Introduction

Inflammatory gallbladder disease is one of the most common abdominal surgical disorders in emergency departments [1]. The incidence of acute cholecystectomy (AC) corresponds to the incidence of acute appendicitis in adults, while the incidence of AC in pediatric patients is significantly lower [2,3].

For AC, laparoscopic cholecystectomy (LC) is the gold-standard treatment [4]. However, while minimally invasive surgery is common worldwide, the incidence of complications, especially for common bile duct injury, is increased in laparoscopic and robotic cases, compared to open surgery, particularly in complex cases [5,6].

The optimal time to perform LC is still a matter of debate. In 2020, the World Society of Emergency Surgery recommended LC as the first line of treatment and that early laparoscopic cholecystectomy (ELC) should be the standard of care whenever possible, confirming the pivotal role of this surgery [7]. However, the most common and widely validated guidelines on AC are the Tokyo Guidelines 2018 (TG18), which eliminated the time limit (<72 h) and expanded the surgical indication to severe AC. The optimal timing is as soon as possible; for AC, LC is considered ELC when performed 24–72 h after the onset of symptoms and delayed cholecystectomy (DLC) after 6–8 weeks. Patients with AC should undergo LC during the initial hospital admission [3]. However, the 72 h rule is still the gold standard, though no difference was found in patients operated on 7 days after the onset of symptoms [7].

Nevertheless, LC within 24 h of hospital admission has been shown to be superior to the conservative approach when concerning morbidity and cost [3]. There were statistically significant reductions in mortality, total complication rate, bile duct leaks and injuries, wound infections, conversion rate, length of hospital stay, and blood loss associated with ELC [8,9].

Non-operative management followed by planned LC is associated with a doubling of the risk of bile duct injury or bile leak, which creates a strong argument for ELC in AC [3].

Although the superiority of ELC over DLC has been proven, it is not always implemented. For example, in the USA, only 30% of individuals with AC undergo LC during its acute phase, and in the UK, only 20% of surgeons perform LC for AC [10].

One reason for failing to implement ELC for AC is the usual delay in access to emergency operating theaters and the belief that AC is not a surgical emergency. In addition, the lack of knowledge of new guidelines and the absence of a standardized internal management flowchart for AC may lead to increased preoperative times and rates of DLC. The preoperative phase remains the most significant driver of the cost differential between urgent and elective cases. The average postoperative stay following LC is 24 h, emphasizing the lack of advantages to long preoperative hospitalization [11].

Considering the importance of preoperative factors and their link to postoperative surgical outcomes, the aim of this study is to evaluate the influence of preoperative waiting time (WT) on the postoperative length of stay (LOS), considering it not only a measure of postoperative complications but also the time required for recovery.

## 2. Materials and Methods

### 2.1. Patients

For this study, we selected patients from two emergency surgery departments in two different public university hospitals: Fondazione IRCCS Policlinico Ca’ Granda in Milano and Cattinara University Hospital in Trieste. All adult patients (>18 years old) admitted to the two centers and submitted to surgery for acute cholecystitis between June 2021 and September 2022 were included.

This study complied with the Declaration of Helsinki, and all data were anonymized when uploaded to our secure study database.

### 2.2. Study Design

We conducted a prospective, observational, non-randomized, multicenter cohort study. We enrolled all consecutive patients admitted with acute cholecystitis between June 2021 and September 2022 and followed those patients for 10 days post-admission (up to 10 October 2022). Acute cholecystitis was graded using the Tokyo Guidelines grading system, which provided a uniform method [12]. The grading system uses clinical, radiographic, operative, and pathologic criteria to assign an incremental ordinal severity score from 1 to 3. Furthermore, this study complied with the Strengthening the Reporting of Observational Studies in Epidemiology (STROBE) guidelines.

The inclusions criteria included adult (>18 years) patients admitted to the emergency department for AC and submitted to LC.

Exclusion criteria were the following:

- Patients not suitable for surgery due to comorbidities or a poor general condition were excluded. We used the WSES classification to define the patients considered unfit for surgery [13]: “Unfit for surgery. Patients that satisfy at least one of the following criteria: age ≥ 80 years, ASA ≥ grade 3, age-adjusted CCI score ≥ 4, and/or Karnofsky score associated with severe or end-stage liver cirrhosis, degenerative or malignant disease, hematologic disorders, or any other severe disease/comorbidity preventing the possibility to undergo surgical intervention with a reasonable chance to survive up to a reasonable recovery”.
- Patients with a concomitant oncological disease (e.g., gallbladder tumor, tumor in the head of the pancreas) were excluded.
- Patients subjected to a bridge treatment, such as percutaneous gallbladder drainage or endoscopic ultrasound gallbladder drainage, were excluded.

Preoperative workout was standardized, as reported below.

Blood tests and abdominal ultrasonography were performed for all patients submitted to the ED for AC. In the case of a normal liver and pancreatic function blood test, patients were evaluated by the emergency surgery team (head surgeon, anesthesiologist, and scrub nurse) and scheduled for surgery according to operation room availability. When liver function tests were elevated, the risk of choledocholithiasis was evaluated using criteria from the European Society of Gastrointestinal Endoscopy [13,14]. Based on the patient’s clinical condition and the availability of resources, they were then scheduled for either MR cholangiography or endoscopic ultrasound, followed by surgery.

Patients admitted to the ED with acute cholecystitis associated with pancreatitis were scheduled for surgery only after a decrease in serum pancreatic enzyme levels [15].

Data on intraoperative and postoperative complications were collected, and the severity of postoperative complications was graded according to the Clavien–Dindo classification [16].

### 2.3. Study Outcome

The primary outcome of this study was the analysis of the factors that affect the length of stay (LOS). The secondary outcomes were the severe complications within 30 days, as defined by a Clavien–Dindo classification grade of 3 to 5 (reoperation, reintervention, unplanned admission to intensive care unit, organ support requirement, or death).

### 2.4. Surgical Procedure

All surgical procedures began with a minimally invasive approach. The patient was placed in a supine position, with the first surgeon located between his legs, the so-called French position [17,18]. The reason for conversion was a first-operator choice, which depended on multiple factors, such as a difficult anatomy and extensive adhesions due to previous surgery and bleeding. The grading of the cholecystitis was defined by the Tokyo Guidelines [12]. All specimens were sent to the department of pathology for a pathological exam. Abdominal drainage placement was a first-surgeon choice, mainly driven by the complexity of the surgery. Drainage was removed 24/48 h after surgery, according to the amount and characteristics of the output. The patient was discharged 24/48 h after surgery or after the removal of the drainage, along with a normal or improved blood test and in accordance with their clinical condition (pain control and restart of oral feeding) [19].

### 2.5. Follow-Up

All patients were scheduled for an outpatient consultation 10 days after surgery, where they were submitted to a clinical examination and the removal of surgical stitches. No further examination was scheduled.

### 2.6. Statistical Analysis

After being selected according to the inclusion criteria, patients were grouped based on the timing of the cholecystectomy: early (within 48 h of admission or a decrease in liver function tests) vs. late (after 48 h of admission or a decrease in liver function tests) cholecystectomy. All data were tested for a normal distribution via a Shapiro–Wilk test. The descriptive results for continuous, normally distributed variables were presented as means and standard deviations (SDs), and for continuous, non-normally distributed variables, as medians and interquartile ranges (IQRs). Categorical variables were presented as counts and percentages. Continuous, normally distributed variables were compared using a Student’s *t*-test, while continuous, non-normally distributed variables were compared using a Wilcoxon test. Fisher’s exact test was used for categorical variables, as appropriate (see footnotes Table 1) [20]. In all analyses, a two-tailed *p*-value of less than 0.05 was considered statistically significant. Statistical analysis was performed by first comparing patients submitted to ELC and DLC. Then we ran first univariate and then multivariate analyses using postoperative LOS, categorized as ≥3 days vs. <3 days, as variables. Analyses were conducted with the statistical software R 4.1.1 (R Foundation for Statistical Computing, Vienna, Austria) using the jamovi package.

## 3. Results

A total of 170 patients, mostly females (52.4%), were enrolled in our study, with a median age of 64 years (IQR 50.0–78.0). The median Charlson Comorbidity Index was 2 (IQR 1–4). The median preoperative time was 1 day (IQR 0–3.0). The most common Tokyo Guidelines severity gradings were II and III. Postoperative complications occurred in 37 patients (21.8%), with the following Clavien–Dindo classification grades: 0 (1.2%), 1 (72.4%), 2 (20.0%), 3 (3.5%), and 4 (2.9%). The median LOS was 6.00 days (2.00–40.00), while the median WT was 1.00 (0.00–22.00). A comparison of ELC and DLC patients is presented in Table 1.

A univariate analysis was performed (see Table 2) using LOS as the dependent variable (considering 3 days of hospitalization as a cut-off, as described in the Materials and Methods). Only variables with a *p*-value < 0.05 were included in the multivariate analysis. In the multivariate analysis, only variables with a *p*-value < 0.05 were considered statistically significant.

## 4. Discussion

Our study shows that LOS depends on preoperative WT. The diagnosis that mostly led to surgery was acute cholecystitis, which is in line with current literature, where ACC (acute calculous cholecystitis) is described as the most common entity in the hepatobiliary system, and cholecystectomy is the most common surgical intervention [21].

Regarding LOS, we considered a cut-off of 3 days. This finding is not uniformly supported in literature, as a standardized LOS has not been universally defined for patients undergoing cholecystectomy. We chose this cut-off based on our series of patients and a review of the current literature. The median LOS of our series of patients treated with LC for ACC was 3 days for those with an uneventful postoperative course and 10 days for those with postoperative complications. The choice of the cut-off was also based upon one of the largest series of patients treated with LC for ACC: Zafar et al. included 95,523 patients in their study, reporting a median LOS between 2 and 4 days [22]. Also, in the series of Lecoq et al., a postoperative LOS > than 3 day was considered a prolonged postoperative course [11].

In our study, the median LOS was 6 days, which is quite long compared to the median time reported in literature. This may be explained by the age of the population (median of 64 years) and the high rate of Tokyo Guidelines severity grades II and III. The high median age reflects the high incidence of comorbid patients who must face a more complex postoperative course, due to the treatment of concomitant diseases. Age negatively influences ERAS protocols, making the postoperative course more challenging and complex. Patients with high Tokyo Guidelines severity grades may present an impaired general condition associated with sepsis. In these cases, postoperative admission to the ICU may lead to a longer postoperative course and increases the risk of nosocomial infections.

There is no standard definition of ELC and DLC in literature, and there are no specific cut-offs. In our study, we considered ELC to be LC performed within 48 h of ER access or the normalization of blood tests in the case of acute pancreatitis and DLC for those over 48 h from the diagnosis of acute biliary disease.

The timing of surgical interventions has traditionally been determined by the duration of symptoms, but recent evidence supports earlier surgery as a safe practice, regardless of the time from the onset of symptoms [23]. The duration of symptoms is not an independent prediction and should not influence a surgeon’s decision to perform a cholecystectomy [9]. In our series, the period from onset was longer in the DLC group, suggesting that patients with a longer period from onset are more likely to be treated conservatively initially and studied to assess further details before surgery but eventually, it was not statistically correlated to prolonged LOS.

Ideally, surgery should be performed in the “golden 72 h” [24]. The “72 h rule” is still commonly referred to, while in other reviews, 7 days is considered an acceptable time. Several cut-offs are considered in current literature, including the 48 h cut-off, even if it is not suggested in most papers [25]; however, we wanted to consider a stricter timing to obtain the most stringent evidence and endorse more rigid cut-offs to lead patients to ever-earlier operations.

The 2020 WSES guidelines on ACC recommend ELC as soon as possible, within 7 days of hospital admission and 10 days from the onset of symptoms, even for high-risk patients [26]. Studies investigating the “72 h rule” demonstrated that ELC within 72 h led to significantly improved outcomes, compared to surgery after 72 h [24]. Even if ELC within a 72 h window is optimal, patients operated upon after this time still benefit from the surgery, compared to those undergoing a delayed operation.

The execution of ELC has many advantages: it is associated with fewer intraoperative and postoperative complications, a much shorter LOS, and quick recovery. As has been noted, patients who underwent ELC had significantly fewer major complications [1]. One explanation for such results is that cholecystectomy performed within this timeframe reduces the risk of injury to the structures within Calot’s triangle.

Even if intra- and postoperative complications were similar between the two surgical groups within our study, LOS was significantly shorter in the ELC group. LC performed within 2 days of the presentation of AC yielded the best outcomes and lowest costs [27].

Even if we did not perform an economic analysis of the impact of ELC on hospital costs, a reduced LOS may be considered a surrogate of the overall cost of the procedure. Less postoperative complications and a shorter LOS equate to a faster recovery of patients and lower costs of hospitalization. Data from literature support the economic benefit of ELC: Gutt et al. showed a 46% increase in cost for patients receiving cholecystectomy 24 h after presentation [28]. This demonstrates that the ability to provide earlier surgery could be beneficial to hospitals in terms of operative time and total length of stay, as well as improve patients’ healthcare experiences, reducing the overall burden of disease. The economic benefits of earlier LCs may also be extended directly to patients: EC was associated with fewer days lost from work, compared to delayed LC [28]. ELC is shown to be less expensive and results in better QALY (quality-adjusted life year) scores than DLC, which indicates an improved quality of life for early group patients, compared to delayed patients [29]. In pancreatitis, ELC is also recommended. Patients with mild gallstone pancreatitis should have a cholecystectomy during index admission within 48 h of arrival, regardless of the resolution of abdominal pain or normalization of serum amylase levels. LC within 48 h of admission is safe and decreases hospital length of stay [30]. The PONCHO trial demonstrated the superiority of same-admission cholecystectomy over interval cholecystectomy for mild gallstone pancreatitis in terms of the risk of recurrent pancreatitis [31].

When ERCP is performed, same-admission cholecystectomy is still advised. Cholecystectomy may be performed as early as the second hospital day if the patient is clinically improving. On the other hand, unlike what might be expected, LOS is not significantly related to the conversion rate.

The conversion rates for LC in AC in literature are reported to be up to 22.5% [32]. In our study, the conversion percentages were found to be in line with those reported. In Milan, it was 2.5%, and in Trieste, it was 13.5%. The different rates of conversion may be explained by the performance of difficult cholecystectomies during the night and the absence of a dedicated emergency surgical team unit in the Cattinara Hospital. The conversion rate was higher when cholecystectomy was delayed by more than 24 h after ERCP [33], and acute cholecystitis is a well-recognized predictor of conversion [34], as wall thickness (>4–5 mm), a fibrotic gallbladder, and past history are predictors for a difficult LC [35]. Regarding other indexes, the median duration of the two interventions was the same (115 min for ELC and DLC), with a higher conversion rate in ELC (10.1%), as well as a major use of drainage (79.8%, *p* = 0.001).

Considering the overall complication rate, studies show that occurrences of total biliary complications are significantly lower with ELC, demonstrating that ELC for AC is not associated with increased severe perioperative complications [36]. Our results showed that the most severe complications (Clavien–Dindo 3 and 4) occurred in the ELC group, but this was not statistically significant.

WT is related to the risk of postoperative complications, even if not significantly. This suggests that leaving more time between the diagnosis and surgery allows for the inflammation to mature, making the intervention more complex and increasing the risk of complications [37]. These data are supported by our findings, in which WT is greater in DLC than ELC, leading to a greater LOS in DLC, compared to ELC. The reasons for a delayed WT are both patient-related and hospital-related. Complex patients with comorbidities require more preoperative tests and, consequently, a longer WT. Hospital and emergency department congestion may delay access to an OR. In both cases, the inflammation process that affects the gallbladder may increase the complexity of the surgery and LOS. A decreased LOS and higher bed turnover will likely have the added benefit of decompressing already extended emergency rooms, recovery rooms, and intensive care units.

Even including data about systemic and local inflammation, such as leukocyte PCR and the Tokyo Guidelines grading system, WT remains the most predictive factor of postoperative length of stay. This may be explained by the fact that WT is an indicator that includes all the preoperative factors that influence the outcome of patients with AC. Several studies have reported the impact of preoperative waiting time on postoperative complications, such as the paper of Lai et al. on acute appendicitis. A long preoperative time for high-risk patients has also been related to increased postoperative complications [37,38,39].

Our study has several limitations. Firstly, it is a non-randomized study conducted in two centers. The clinical presentation of patients, surgical procedures, and laparoscopic skills may differ between the two centers, and this may add some bias. The center in Trieste is a general surgery unit dedicated to elective cases, so the majority of emergency cases are managed in the evening or during the night, while the center in Milan is a dedicated emergency unit with a specific commitment to acute care surgery. This in itself may lead to selection bias. Secondly, the retrospective method to select patients and the selection of only surgical cases may also introduce some important selection bias. Unfortunately, in both institutions, several patients are admitted to other departments other than the surgical one, and patients treated with non-operative management may often be excluded. The LOS variable may also be influenced by pre-existing conditions, such as patient comorbidities and previous visits to the emergency department. Even different treatment flowcharts in the two hospitals may lead to different postoperative management courses and, consequently, different LOSs for similar patient patterns.

## 5. Conclusions

Our study provides further confirmation that WT significantly influences LOS, emphasizing how making patients wait longer for an operation (whatever the initial diagnosis) forces them to be hospitalized for longer. Our data show the importance of preoperative WT in determining the surgical outcome of patients submitted to emergency cholecystectomy. This implies the necessity of developing a dedicated decisional and management flowchart for these patients to better allocate resources for their effective treatment.

## Figures and Tables

**Table 1:** Comparison between late cholecystectomy (DLC) vs. early cholecystectomy (ELC).

	DLC		ELC		*p*	Test
n	71		99			
age Mdn [IQR]	57.00	[48.50, 76.50]	67.00	[52.00, 78.00]	0.145 ^a^	non-norm
sex = female %	42	(59.2)	47	(47.5)	0.178 ^b^	
preop WT Mdn [IQR]	3.00	[2.00–6.00]	0.00	[0.00, 1.00]	**<0.001** ^a^	non-norm
leukocytes Mdn [IQR]	11.00	[8.07, 14.48]	10.85	[9.02, 15.20]	0.770 ^a^	non-norm
PCR Mdn [IQR]	0.98	[0.37, 6.40]	0.70	[0.38, 1.78]	0.651 ^a^	non-norm
Total Bilirubin Mdn [IQR]	0.80	[0.50, 1.42]	0.89	[0.58, 1.39]	0.905 ^a^	non-norm
TG %					**<0.001** ^b^	
1	47	(69.1)	41	(43.2)		
2	18	(26.5)	54	(56.8)		
3	3	(4.4)	0	(0.0)		
Days Onset Mdn [IQR]	8.00	[5.00, 13.00]	4.00	[2.00, 6.50]	**0.001** ^a^	non-norm
CCI Mdn [IQR]	1.00	[0.50, 3.50]	2.00	[1.00, 4.00]	**0.036** ^a^	non-norm
Duration Mdn [IQR]	115.00	[75.00, 135.00]	115.00	[85.50, 140.00]	0.269 ^a^	non-norm
conversion (%)	4	(5.6)	10	(10.1)	0.446 ^b^	
intra operative complications (%)	8	(11.3)	6	(6.1)	0.350 ^b^	
Drainage (%)	38	(53.5)	79	(79.8)	**0.001** ^b^	
post operative complications (%)	15	(21.1)	22	(22.2)	1000 ^b^	
Clavien–Dindo (%)					0.139 ^b^	
0	2	(2.8)	0	(0.0)		
1	51	(71.8)	72	(72.7)		
2	16	(22.5)	18	(18.2)		
3	2	(2.8)	4	(4.0)		
4	0	(0.0)	5	(5.1)		
LOS days (Mdn [IQR])	9.00	[6.00, 11.00]	5.00	[3.50, 7.00]	**<0.001** ^a^	non-norm

PCR: protein C reactive. WT: preoperative waiting time; TG: Tokyo Guidelines grade; CCI: Charlson Comorbidity Index; LOS: length of stay, Mdn: median, IQR: interquartile range. *p*-values < than 0.05 are in bold. ^a^ Wilcoxon tests, ^b^ Fisher’s exact test.

**Table 2:** Univariate and multivariate analyses.

			Univariate		Multivariate	
	LOS ≤ 3	LOS > 3	OR (95% CI)	*p* Value	OR (95% CI)	*p*-Value
age	58.6 (17.6)	64.4 (16.9)	1.02 (1.00–1.04)	*p* = 0.040	1.10 (1.00–1.24)	*p* = 0.071
sex	26 (32.1)	55 (67.9)	-		-	
	30 (33.7)	59 (66.3)	0.93 (0.49–1.76)	*p* = 0.824	-	
Time to surgery	108.8 (32.5)	120.4 (50.3)	1.01 (1.00–1.01)	*p* = 0.119	-	
Drainage	22 (41.5)	31 (58.5)	-		-	
	34 (29.1)	83 (70.9)	1.73 (0.88–3.41)	*p* = 0.111	-	
postoperative complications	53 (39.8)	80 (60.2)	-		-	
	3 (8.1)	34 (91.9)	7.51 (2.53–32.29)	*p* = 0.001	13.90 (0.79–972.98)	*p* = 0.142
WT	0.6 (0.8)	2.9 (3.5)	2.21 (1.61–3.25)	*p* < 0.001	8.08 (1.65–77.18)	*p* = **0.033**
ELC	10 (14.1)	61 (85.9)	-		-	
	46 (46.5)	53 (53.5)	0.19 (0.08–0.40)	*p* < 0.001	10.89 (0.53–370.60)	*p* = 0.148
CCI	2.0 (1.8)	2.8 (2.2)	1.19 (1.02–1.42)	*p* = 0.034	0.34 (0.09–0.99)	*p* = 0.079
leukocytes	12.5 (6.5)	12.3 (5.7)	1.00 (0.91–1.09)	*p* = 0.926	-	
PCR	5.6 (9.8)	4.5 (7.7)	0.99 (0.93–1.05)	*p* = 0.623	-	
Total bilirubin	0.7 (0.2)	2.2 (2.8)	4.60 (1.51–26.33)	*p* = 0.043	6.99 (1.16–220.45)	*p* = 0.176
TG	28 (31.8)	60 (68.2)	-		-	
2	26 (36.1)	46 (63.9)	0.83 (0.43–1.60)	*p* = 0.568	-	
3	1 (33.3)	2 (66.7)	0.93 (0.09–20.58)	*p* = 0.956	-	
Days Onset	4.7 (4.3)	9.7 (7.7)	1.26 (1.09–1.52)	*p* = 0.005	1.11 (0.98–1.38)	*p* = 0.202

PCR: protein C reactive. WT: preoperative waiting time; TG: Tokyo Guidelines grade; CCI: Charlson Comorbidity Index; LOS: length of stay ELC: early cholecystectomy. *p*-values < than 0.05 are in bold.

## Data Availability

Raw data were generated at Department of Emergency Surgery, Fondazione IRCCS Ca’ Granda Ospedale Maggiore Policlinico, Via Francesco Sforza 35, 20122 Milano, Italy and Department of General Surgery, Cattinara University Hospital, Azienda Sanitaria Universitaria Giuliano Isontina (ASU GI), Trieste University, Strada di Fiume 447, 34149 Trieste, Italy. Derived data supporting the findings of this study are available from the corresponding author M.C. on request. at https://www.mdpi.com/ethics.
